# Natriuretic peptide receptor A as a novel target for cancer

**DOI:** 10.1186/1477-7819-12-174

**Published:** 2014-06-03

**Authors:** Jia Zhang, Zhilong Zhao, Jiansheng Wang

**Affiliations:** 1 Department of Thoracic Surgery 2, First Affiliated Hospital of Xi’an Jiaotong University, Xi’an, Shaanxi 710061, China

**Keywords:** NPR-A, Cancer, Target

## Abstract

The receptor for the cardiac hormone atrial natriuretic peptide (ANP), natriuretic peptide receptor A (NPR-A), has been reported to be expressed in lung cancer, prostate cancer and ovarian cancer. NPR-A expression and signaling is important for tumor growth; its deficiency protects C57BL/6 mice from lung, skin and ovarian cancers. This suggests that NPR-A is a new marker and a new target for cancer therapy. Recently, NPR-A has been demonstrated to be expressed in pre-implantation embryos and in embryonic stem cells, which has a novel role in the maintenance of self-renewal and pluripotency of embryonic stem cells. A nanoparticle-formulated interfering RNA for NPR-A attenuated B16 melanoma tumors in mice. Ectopic expression of a plasmid encoding NP73-102, the NH2-terminal peptide of the ANP prohormone which downregulates NPR-A expression, also suppressed lung metastasis of A549 cells in nude mice and tumorigenesis of Line 1 cells in immunocompetent BALB/c mice. These results suggest that NPR-A is involved in tumorigenesis and a new target for cancer therapy. This review focuses on structure, abnormal functions and carcinogenic mechanisms of NPR-A to investigate its role in tumorigenesis.

## Review

### Introduction

Natriuretic peptide receptor A (NPR-A) is the receptor for atrial natriuretic peptide (ANP) and brain natriuretic peptide (BNP). ANP and BNP, members of the natriuretic peptide family, have been extensively studied for their functions in regulating blood pressure. Both ANP and BNP signal through NPR-A by increasing cGMP and activating cGMP-dependent protein kinase G (PKG). Activated PKG in turn upregulates expression of genes encoding ion transporters and transcription factors, which together affect cell growth, apoptosis, proliferation, and inflammation [1-3]. The human NPR-A gene is located on chromosome 1q21-22 and consists of 22 exons and 21 introns within 16 kb. NPR-A and/or its mRNA is expressed in kidney, lung, adipose, adrenal, brain, heart, testis, and vascular smooth muscle tissue [4-7]. Recent research suggests, however, that NPR-A expression and signaling is important for tumor growth. NPR-A deficiency protected C57BL/6 mice from lung, skin, and ovarian cancers. Furthermore, a nanoparticle-formulated interfering RNA for NPR-A attenuated B16 melanoma tumors in mice. Ectopic expression of a plasmid encoding NP73-102, the NH2-terminal peptide of the ANP prohormone which downregulates NPR-A expression, also suppressed lung metastasis of A549 cells in nude mice and tumorigenesis of Line 1 cells in immunocompetent BALB/c mice. These results suggest that NPR-A is involved in tumorigenesis and a new target for cancer therapy [8,9]. This review focuses on structure, abnormal functions and carcinogenic mechanisms of NPR-A to investigate its role in tumorigenesis.

### The structure of natriuretic peptide receptor A

The human NPR-A gene is approximately 16 kb, contains 22 exons and 21 introns, and is located on chromosome 1q21–22 that encodes a 1,061-amino acid peptide [5,10]. The NPR-A gene product is abundantly expressed in the vascular system, kidneys, and adrenal glands [11]. NPR-A has been cloned and sequenced from rat brain [12,13], human placenta [14], and mouse testis [15]. The general topological structure of NPR-A is consistent with that in the GC receptor family, containing at least four distinct regions: an extracellular ligand-binding domain (ECD), a single transmembrane spanning region, an intracellular protein kinase-like homology domain (protein-KHD), and a GC catalytic domain [15,16]. Activation of the NPR-A receptor requires ANP binding to the ECD and phosphorylation of up to six residues within the KHD [17]. After ANP binding to NPR-A, activation of the intrinsic GC leads to an intracellular increase in cGMP which in turn can activate different downstream effectors involved in cell growth, apoptosis, proliferation and inflammation, through the activation of cGMP-dependent PKG [3].

The ECD contains a highly conserved chloride-binding site near the ECD dimerization interface [18,19]. The ECD also contains in its juxtamembrane region a highly conserved structural motif, referred to as the receptor-GC signaling motif [20]. Single-residue mutations in this motif either render the receptor unresponsive to ligand binding or cause constitutive activation of GC activity [21], suggesting that this conserved structure plays a critical role in transmembrane signal transduction. The ECD contains three disulfide bonds, Cys60–Cys86, Cys164–Cys213 and Cys423–Cys432, in a 1–2, 3–4 and 5–6 linkage pattern [22]. Of these, the disulfide bond Cys60–Cys86 occurs in the chloride-binding site, and the disulfide bond Cys423–Cys432 occurs in the juxtamembrane receptor-GC motif. Both disulfide bonds are conserved among the A-type and B-type natriuretic peptide receptors. The ECD also contains five N-linked oligosaccharides [23]. Correct glycosylation is essential for expression of functional NPR-A: deletion of any one of the five glycosylation sites by mutagenesis reduces or abolishes NPR-A expression [24]. On the other hand, deglycosylation of the native or expressed ECD with endoglycosidase F2 or H has no effect on ANP binding [25]. Together, these findings suggest that glycosylation is essential for folding or transport of the nascent receptor polypeptide to the cell membrane, but that, once the active receptor is formed, the glycosyl moieties are not involved in ANP binding. This notion is consistent with the crystal structure of the ANP–ECD complex [18], which shows that glycosyl moieties or the glycosylation sites are located away from the ANP-binding site.

The KHD contains an approximately 280-amino acid region that immediately follows the transmembrane spanning domain of the receptor. The KHD of NPR-A is more closely related to protein tyrosine kinases than protein serine/threonine kinases. In fact, it is largely similar to the protein kinase domain of the platelet-derived growth factor receptor with approximately 31% amino acid sequence identity between the comparable regions of the kinase domains [5,12,15]. It has been demonstrated that the KHD of NPR-A serves an important mediatory role in transducing the ligand-induced signals to activate the GC catalytic domain of the receptor [16,26,27]. It has been suggested that an intervening step involving the KHD is necessary for the cyclase catalytic activation process [24,28,29]. Several studies have suggested that ATP serves as an intracellular allosteric regulator of the KHD for the activation of NPR-A [27,30-33]. ATP is thought to function by interacting with the KHD because this region contains a glycine-rich nucleotide-binding motif and has been postulated to provide an ATP-regulatory module for ANP signaling [27,34]. It has been also suggested that binding of ANP to NPR-A activates ATP binding to the KHD in the intracellular cytoplasmic space, which, in turn, activates the GC catalytic domain of the receptor [27,28,35]. Indeed, previous studies as well as recent data have indicated that the KHD seems to be important for ANP-dependent activation of NPR-A [36,37]. However, the exact mechanisms of activation and relay of signals from the KHD to the GC catalytic active site of the receptor remain to be established.

The GC catalytic domain of NPR-A contains an approximately 250-amino acid region that constitutes the catalytic active site of the receptor [38-40]. The transmembrane GC receptors contain a single cyclase catalytic active site per polypeptide molecule; however, based on the structure modeling data [41] two polypeptide chains seem to be required to activate NPR-A, and the receptor functions as a homodimer [42-44]. Initially, the dimerization region of the receptor was believed to be located between the KHD and GC domain that has been suggested to form an amphipathic alpha helix structure [45]. It has been reported that the crystal packing of the extracellular ligand-binding domain of NPR-A contains two possible dimer pairs, the head-to-head and tail-to-tail dimer pairs associated through the membrane-distal and membrane-proximal subdomains, respectively [46]. The tail-to-tail dimer of NPR-A has been proposed previously [41]. The crystal structure of NPRC has also suggested that NPRC is dimerized in head-to-head configuration bound with ligand CNP [47]. It has been previously proposed that the head-to-head dimer may represent the latent inactive state and the tail-to-tail dimer could represent the hormone-activated state [48]. It has also been indicated that ligand-dependent activation of NPR-A stabilizes a membrane-distal dimer interface of this receptor protein suggesting that ANP binding stabilizes the NPR-A dimer with more stringent spacing at the dimer interface [49]. However, more recently, the results of site-directed mutagenesis and chemical modification studies suggested that head-to-head dimer structure reflects the physiological dimer structure of NPR-A [46].

### Natriuretic peptide receptor A and cancer

Almost all studies of NPR-A expression have focused on heart, kidney or other tissues. In 1993, Ohsaki and colleagues reported that functional ANP A receptors (NPR-A) are expressed in human small-cell lung cancer cell lines and Hela cells for the first time [50]. NPR-A mRNA was identified by Southern blot analyses of PCR products and RNase protection assays using poly (A) (+)-selected RNA. Vesely reported natriuretic peptides including ANP inhibit proliferation of various cancer cells and tumor growth, and NPR-A is expressed in various cancer cells [51], but he did not investigate the direct role of NPR-A in tumorigenesis. Recently the direct role of NPR-A signaling in tumorigenesis has been clarified by animal model, interfering RNA, Western blot and immunohistochemistry [8,9].

#### Natriuretic peptide receptor A and lung cancer

In 1993, NPR-A mRNA was identified by Southern blot analyses of PCR products and RNase protection assays using poly (A) (+)-selected RNA, and it was first reported expressed in human small-cell lung cancer cell lines [50]. Western blots results also show that NPR-A is present in human small-cell lung cancer cells [52]. Kong and colleagues [8] also found that NPR-A is expressed at a higher level in lung carcinoma (A549 and LLC1). They also clarified the direct role of NPR-A in lung cancer [8]. It has been shown NPR-A deficiency decreases lung inflammation. The data from animal models that compared the lungs of mice deficient in NPR-A (NPR-A^-/-^) with those of wild-type mice after immunization with intraperitoneal ovalbumin and subsequent challenge with intranasal ovalbumin showed that C57BL/6 wild-type mice had substantially higher inflammation, blocked airways, and goblet cell metaplasia than did NPR-A^-/-^mice. Bronchoalveolar lavage fluid from NPR-A^-/-^mice had significantly reduced levels of the inflammatory cytokines IL-4, IL-5, and IL-6 relative to those in wild-type mice. Using the Lewis lung carcinoma model, compared to C57BL/6 wild-type mice, the tumor in NPR-A^-/-^mice gradually shrank. This indicates that NPR-A deficiency significantly protects mice from lung tumorigenesis and lung tumor progression. NP73-102 nanoparticles were investigated in the suppression of lung cancer tumorigenesis [8]. Using a soft agar assay, A549 cells were transfected with pVAX1, pANP, or pNP73-102, and *in vitro* results from the soft agar assay indicated that cells transfected with pNP73-102 exhibited significantly decreased colony formation compared to that of non-transfected cells or cells transfected with pVAX1. Using a nude mouse model, mice were given 5 × 10^6^ A549 cells intravenously and weekly instillations of PBS (control) or nanoparticles carrying pNP73-102 or pVAX1. Three weeks later, mice were sacrificed and lung sections were stained with hematoxylin and eosin and examined for lung nodules. Control animals receiving only PBS showed nodules and tumors, whereas the NP73-102-treated group had no tumors. In the BALB/c mice tumor model, those treated with pNP73-102 nanoparticles injected weekly showed significantly reduced tumor burden compared to those treated with PBS or pVAX1 control vector [8]. TUNEL assay, Western blot of the cleavage of caspase 3 substrates, and PARP show that NP73-102 induces apoptosis of A549 adenocarcinoma. Microarray analysis of gene expression of A549 cells after transfection with pNP73-102 show that pNP73-102 significantly altered the expression of a number of genes. The upregulated genes were predominantly from the family of interferon-regulated genes or related signal transduction pathways, and the downregulated genes included some involved in inflammation. This indicates that NP73-102 has anti-inflammatory properties.

To investigate the signal pathway in tumor suppression in NPR-A deficient mice, whole proteins were extracted from lungs of wild-type and NPR-A^-/-^mice and then probed using primary antibodies against p50, p65, phospho-p50, and phospho-p65. No significant difference in NF-κB expression in the lungs was abserved between wild-type and NPR-A^-/-^mice. However, the level of the activated form of NF-κB, phospho-NF-κB (both phospho-p65 and phospho-p50) was decreased in NPR-A^-/-^mice. These results indicate that the role of NPR-A in lung inflammation may involve NF-κB activation. Immunohistochemistry analysis results from the expression of pRb in the lungs of wild-type C57BL/6 and NPR-A^-/-^mice revealed that NPR-A deficiency induced overexpression of pRb. Western blot results show that the expression of vascular endothelial growth factor was decreased in the lungs of NPR-A-deficient mice. Superarray analysis indicates that the expression of hexokinase 2, glycogen synthase 1, and matrix metallopeptidase 10 were downregulated in the lungs of NPR-A^-/-^mice, while the expression of cellular retinol binding protein 1 was upregulated in the lungs of NPR-A^-/-^mice.

#### Natriuretic peptide receptor A and skin cancer

Kong and colleagues found that NPR-A is expressed at a higher level in melanoma [8]. siNPR-A nanoparticles were investigated in the injected melanoma cells wild-type mice model; the mice were treated twice a week with a cream containing either synthetic siNPR-A, psiNPR-A, or Scr, respectively. A significant reduction in tumor burden was seen in mice treated with siNPR-A (either with synthetic or psiNPR-A) but not those given Scr after 4 weeks [8]. This result indicates that siNPR-A could be used to treat melanomas. Flow cytometry assay results showed that overexpressing pNP73-102 in B16 melanoma could significantly induce apoptosis but not that of normal NIH3T3 cells [8]. TUNEL assay also showed a similar result.

#### Natriuretic peptide receptor A and ovarian cancer

Kong and colleagues found that NPR-A is expressed at a higher level in ovarian cancer (SKOV3 and ID8) [8]. Using an animal model, wild-type and NPR-A-deficient C57BL/6 mice were injected with ovarian cancer cells and monitored weekly for tumor growth; by week 8, all mice from the wild-type group developed solid tumors, but no tumors were observed in NPR-A-deficient mice [8]. This indicates NPR-A deficiency could inhibit the growth of ovarian cancer cells.

#### Natriuretic peptide receptor A and prostate cancer

In 2005 Western blots revealed that for the first time NPR-A was present in prostate cancer cells [53]. Kong and colleagues found that NPR-A is expressed at a higher level in prostate cancer cells (DU145) [8]. Using Western blot and reverse transcriptase PCR, Wang and colleagues [9] demonstrated that NPR-A is abundantly expressed in tumorigenic mouse and human prostate cells, but not in non-tumorigenic prostate epithelial cells. Immunohistochemistry of tissue microarray showed NPR-A expression is positively correlated with clinical staging. siNPR-A or iNPR-A could induce apoptosis in Pca cells by the downregulation of NPR-A. This effect was linked to NPR-A-induced expression of macrophage migration inhibitory factor (MIF), and it could be significantly reduced by siNPR-A. Animal models show prostate tumor cells implanted into mice deficient in NPR-A failed to grow, and iNPR-A treatment could reduce the tumor burden and MIF expression in TRAMP-C1 xenografts. It was shown that NPR-A expression is correlated with MIF expression during PCa progression by the TRAMP spontaneous PCa model.

#### Natriuretic peptide receptor A and gastric cancer

Our research mainly focuses on NPR-A and gastric cancer. NPR-A expression was examined by Western blot, immunofluorescence and immunohistochemistry. NPR-A was downregulated by transfection of NPR-A shRNA. We demonstrated that NPR-A is abundantly expressed in human gastric cancer AGS cells, but not in non-tumorigenic epithelial cells. NPR-A expression showed positive correlation with clinical staging and prognosis in gastric cancer patients. Downregulation of NPR-A by siNPR-A induced apoptosis in AGS cells. The mechanism of siNPR-A-induced anti-AGS effects was linked to NPR-A-induced expression of KCNQ1, a K + channel overexpressed in AGS and significantly reduced by NPR-A shRNA.

#### Natriuretic peptide receptor A and other cancers

NPR-A was also present in pancreatic adenocarcinoma cells [54], breast adenocarcinoma cells [55], colon adenocarcinoma cells [56], renal carcinoma cells [57], glioblastoma cells [58], and medullary thyroid carcinoma cells [59]. Vesely’s group studied the effect of four peptides (long-acting natriuretic peptide, vessel dilator, kaliuretic peptide and atrial natriuretic peptide) on the cancer cells; they concluded that the mechanism of decrease in the number of cancer cells was via inhibition of DNA synthesis, mediated in part by cyclic GMP; they did not, however, investigate the role of NPR-A in cancer.

### Carcinogenic mechanism of natriuretic peptide receptor A

Athough Vesely’s group only focused on the effect of natriuretic peptides (including ANP) on the proliferation of various cancer cells and tumor growth and they did not investigate the role of NPR-A in cancer, they showed that these peptides decrease expression of NPR-A [60]. The role of NPR-A in cancer has been clarified by Kong and colleagues using animal models and siRNA [8]. NPR-A-deficient mice showed significantly reduced antigen-induced pulmonary inflammation. NPR-A deficiency could protect C57BL/6 mice from lung, skin, and ovarian cancers. A nanoparticle formulated interfering RNA for NPR-A attenuated B16 melanoma tumors in mice. Ectopic expression of a plasmid encoding NP73-102 could suppress lung metastasis of A549 cells in nude mice and tumorigenesis of Line 1 cells in immunocompetent BALB/c mice by the downregulation of NPR-A expression. Western blot and immunohistochemistry staining indicated that NF-κB was inactivated while retinoblastoma was upregulated in the lungs of NPR-A-deficient mice. The expression of vascular endothelial growth factor was also downregulated in the lungs of NPR-A-deficient mice compared with that in wild-type mice.

Recently it has been demonstrated that NPR-A is expressed in pre-implantation embryos and in embryonic stem cells. Knockdown of NPR-A by RNAi resulted in phenotypic changes, indicative of differentiation, downregulation of pluripotency factors and upregulation of differentiation genes. NPR-A knockdown also resulted in a marked downregulation of phosphorylated Akt [61]. These result from pre-implantation embryos and embryonic stem cells may give us some clues in the study of NPR-A in cancer.

## Conclusion

Although NPR-A has been reported to be expressed in cancer cells for 30 years [50], it was not until 2008 that the role of NPR-A in cancer was studied [8]. Until now, only two papers have clarified the role of NPR-A in cancer [8,9]. Our research mainly focuses on the NPR-A and gastric cancer. We demonstrated that NPR-A is abundantly expressed on human gastric cancer AGS cells, but not in non-tumorigenic epithelial cells. NPR-A expression showed positive correlation with clinical staging and prognosis in gastric cancer patients. Downregulation of NPR-A by siNPR-A induced apoptosis in AGS cells. The mechanism of siNPR-A-induced anti-AGS effects was linked to NPR-A-induced expression of KCNQ1, a K + channel overexpressed in AGS and significantly reduced by NPR-A shRNA. More research needs to focus on the role of NPR-A in cancer, which could clarify the carcinogenic mechanisms of NPR-A more clearly.

From our results and others [8,9], it has been shown that NPR-A is a new target for cancer therapy. More research needs to focus on NPR-A to improve the efficiency of cancer therapy.

## Abbreviations

ANP: atrial natriuretic peptide; BNP: brain natriuretic peptide; ECD: extracellular ligand-binding domain; IL: interleukin; KHD: kinase-like homology domain; MIF: migration inhibitory factor; NPR-A: natriuretic peptide receptor A; PBS: phosphate-buffered saline; PCR: polymerase chain reaction; PKG: protein kinase G.

## Competing interests

The authors declare that they have no competing of interests.

## Authors’ contributions

JZ and ZZ wrote the paper. JW reviewed and edited the manuscript. All authors read and approved the final manuscript.
